# Comparative analysis of physicochemical properties, sensory characteristics, and volatile flavor compounds in five types of potato chips

**DOI:** 10.3389/fnut.2025.1525480

**Published:** 2025-03-19

**Authors:** Qiaozhen Zhang, Yue Sun, Yahui Sun, Cui Guo, Jiayin Zhu, Xinkui Niu, Mingshuang Gao

**Affiliations:** Food Laboratory of Zhong Yuan, Luohe, China

**Keywords:** potato chips, physicochemical properties, sensory characteristics, volatile flavor compounds, gas chromatography-ion mobility spectroscopy, ROAV, correlation analysis

## Abstract

**Introduction:**

Potato chips are the primary product of the potato leisure food market. And the level of consumer preference is strongly influenced by their flavor.

**Methods:**

In this study, five potato chips were compared by combining their physicochemical properties, sensory characteristics, and volatile flavor compounds. The volatile flavor compounds in potato chips were evaluated using gas chromatography-ion mobility spectroscopy (GC-IMS).

**Results and discussion:**

GC-IMS identified 57 volatile flavor compounds, including 17 aldehydes, 9 esters, 10 ketones, 3 pyrazines, 12 alcohols, 3 acids, 1 pyridine, and 2 ethers. Moreover, the aldehydes had the largest relative amount and kind. Seven identical key volatile flavor compounds with ROAV ≥ 1 were examined in five potato chips. In addition, the variety of key flavor compounds with ROAV ≥ 1 was more abundant in Leshi (LS) industrial fresh-cut fried potato chips. While the types of key volatile flavor compounds in Leshi (LS) industrial fresh-cut fried potato chips and other composite potato chips varied, the types of key volatile flavor compounds in composite fried and baked potato chips made with various formulations were consistent. In the sensory evaluation, Shuyuan (SY) industrial composite potato chips and Shiyanshi (SYS) homemade composite baked potato chips were preferred overall. The Leshi (LS) industrial fresh-cut fried potato chips and Kebike (KBK) industrial composite fried potato chips had greater relative levels of harmful factors. But none of the potato chips included trans-fatty acids. The correlation heatmap showed that the harmful factors in potato chips were mainly positively correlated with volatile flavor compounds such as aldehydes and ketones originating from the oxidative degradation of fat.

**Conclusion:**

The study provided a reference for choosing appropriate process conditions in potato chip processing so that the safety of the food can be enhanced while obtaining consumer-preferred food flavors.

## 1 Introduction

The consumer market for potato processing products has been growing in recent years as a result of the growth of the potato industry and ongoing improvements in people’s quality of life. The demand for products has been steadily rising. Additionally, customers have raised their expectations for the kinds and sensory qualities of items made from potato processing (1). The leisure food market is growing quickly in the potato processing industry, and the market is getting bigger. Potato chips, the primary product of the potato leisure food market, are crucial to the growth of the potato business, and the level of consumer preference is strongly influenced by their flavor (2). Potato chips are prepared from fresh-cut potatoes or potato flour by frying or baking, mainly including fresh-cut fried potato chips and composite fried or baked potato chips prepared with different formulations. Fresh-cut fried potato chips are prepared by washing, peeling, slicing, deep-frying, flavoring, and other processes (3), which have obvious potato flavor. Composite fried or baked potato chips are based on potato flour and other raw materials, prepared by mixing, rolling, molding, frying or baking, flavoring, and other processes. The flavor of potato chips mainly comes from the volatile flavor compounds in them. And different formulations and processing methods may affect the composition and content of these volatile flavor compounds. Therefore, analyzing the flavor characteristic components of potato chips is conducive to stabilizing and improving product quality.

Nowadays, the most often used methods for evaluating the volatile taste compounds of potato chips or potatoes are gas chromatography–mass spectrometry (GC–MS), electronic nose, and high-performance liquid chromatography (HPLC) (4). In contrast, gas chromatography-ion mobility spectroscopy (GC-IMS) provides major advantages in determining low-molecular-weight molecules and partially addresses the drawbacks of GC-MS technology (5). This technique combines the separation characteristics of GC and the high sensitivity of IMS without sample pretreatment. With the benefits of a low detection limit, high sensitivity, and a brief analysis period, it can perform trace analysis of gaseous ions (6–8). GC-IMS technology has been widely used in the analysis of volatile flavor compounds and quality detection of various types of food, such as wine (9), steamed bread (10), radish (11), and potato (12). However, the determination of volatile flavor compounds in potato chips using the GC-IMS technique and the comparison of volatile flavor compounds in different types of potato chips have not been reported in the literature. However, there is no research determining volatile flavor compounds in potato chips or comparing volatile flavor compounds in various potato chip kinds using the GC-IMS approach.

In this study, four potato chips with high sales volume in the market (LS industrial fresh-cut fried potato chips, KBK industrial composite fried potato chips, SY industrial composite baked potato chips, and XSC industrial composite baked potato chips) and SYS homemade composite baked potato chips prepared in the laboratory were selected as the study materials. The aromas of the five potato chips were characterized using the GC-IMS technique to construct visual volatile flavor compound fingerprints. And the principal component analysis was performed to reveal the volatile flavor compound differences among the potato chips. The key volatile flavor compounds of the potato chips were screened using the relative odor activity value (ROAV). The differences in physicochemical and sensory properties of different potato chips were also investigated. And the correlation between the physicochemical properties, sensory characteristics, and volatile flavor compounds of potato chips was explored. This study aims to better understand the physicochemical properties, sensory characteristics, and volatile flavor compounds of potato chips, compare the differences between different potato chips, and provide important references for the improvement of potato chip production formulations and processes.

## 2 Materials and methods

### 2.1 Materials

Based on the market research, four commercially available brands of potato chips with high market acceptance within the shelf life were selected, including LS industrial fresh-cut fried potato chips (Leshi, industrial fresh-cut fried potato chips, Bai Shi Food Co., Ltd., Shanghai, China), KBK industrial composite fried potato chips (Kebike industrial composite fried potato chips, Da Li Food Co., Ltd., Quanzhou, China), SY industrial composite baked potato chips (Shuyuan industrial composite baked potato chips, Hao Li You Food Co., Ltd., Langfang, China), and XSC industrial composite baked potato chips (Xiaoshuaicai industrial composite baked potato chips, Luohe Hengda Food Industry Co., Ltd., Luohe, China), as well as SYS homemade composite baked potato chips (Shiyanshi homemade composite baked potato chips, Luohe, China). All potato chips were selected as original flavor chips. Ortho ketones: 2-butanone, 2-pentanone, 2-hexanone, 2-heptanone, 2-octanone, and 2-nonanone Ice acetic acid, trichloromethane, potassium iodide, petroleum ether (30–60°C), hydrochloric acid, ethyl ether, anhydrous ethanol, anhydrous sodium sulfate, iso-octane, methanol, potassium hydroxide, sodium bisulfate, formic acid, n-hexane, ethyl acetate, ammonium sulfate, methylene chloride, acetone, cyclohexane, n-hexane, iso-octane, ammonia, anhydrous ethyl ether, and so forth (Aladdin Biochemical Technology Co., Ltd., Shanghai, China).

### 2.2 Preparation of SYS homemade composite baked potato chips

#### 2.2.1 Formulation

With the increasing awareness of consumers’ healthy diets and living standards, their demand for low-oil, low-harmful-factors, and high-quality baked potato chips is gradually increasing. Therefore, in this study, SYS homemade composite baked potato chips were compared with other types of industrial potato chips on the market so as to provide a reference for the subsequent formulation and preparation process.

Potato flour: 13.32 kg; acetylated double starch adipate: 3.18 kg; potato starch: 0.72 kg; sugar: 2.31 kg; salt: 0.05 kg; sodium bicarbonate: 0.42 kg; phospholipids: 0.36 kg; malic acid: 0.21 kg; phytolipids: 0.33 kg; milk powder: 0.54 kg; maltodextrin: 0.21 kg; potato flavor powder: 0.33 kg; pea fiber: 0.42 kg; water: 9.30 kg.

#### 2.2.2 Preparation process

Preparation of powder material: Sugar and salt were ground into powder. To make sure the sifted powder was free of contaminants, all raw materials were run through a 40-mesh sifter. All powders were weighed in precise proportions, where the added phospholipids were 80% of their total weight. Stirring the mixed powder evenly until there were no obvious lumps. Then placing it in the pasta machine standby.

Preparation of liquid material: Palm oil was kept warm at 60°C. Phospholipids were added as 20% of their total weight and mixed well. The palm oil-phospholipids combination was sheared at high speed at 1,000 r/min for 5 min until a homogenous milky-white emulsion was created when water was gradually added. Additionally, the liquid substance was left to stand until it reached room temperature.

Mixing powder and liquid material: Adding the liquid slowly and evenly to the powder in the mixing machine. After adding, continue mixing for 1–2 min until it was evenly kneaded. And then transferring it to the production workshop for the subsequent process.

Pressing: Pressing the dough through three rolls in total. The thickness of the first roller sheet was 15.0 ± 0.5 mm. The thickness of the second roller sheet was 10.0 ± 0.5 mm. The thickness of the third roller sheet was 5.0 ± 0.5 mm. Lastly, the corrugated rollers pressed the embryo of every potato chip.

Embossing: The embossing shape was heart-shaped and corrugated, with a length of 44.0 ± 1.0 mm, a width of 45.0 ± 1.0 mm, and a thickness of 2.7 ± 1.0 mm.

The first oil spraying: Prior to oil spraying, palm oil was at a temperature of 50°C. The oil weight was 0.22 ± 0.02 g of each piece. As a result, the surface was covered with a uniform layer of oil.

Five-stage roasting: The first roasting was the preheating stage at 210°C. The second roasting was the expansion stage at 250°C. The third roasting was the de-watering stage at 350°C. The fourth roasting was the product finalization stage at 175°C. The fifth roasting was the coloring stage at 135°C. And five-stage roasting for a total of 3 min 50 s.

The second oil spraying: Palm oil was at a temperature of 50°C before spraying oil. The oil weight was 0.13 ± 0.03 g of each piece. As a result, the surface was covered with a uniform layer of oil.

Sprinkling flavor powder: Sprinkling evenly 0.17–0.02 g of per piece on the outer surface of the potato chips.

### 2.3 Measurement of physical and chemical indicators

#### 2.3.1 Measurement of basic components

Fat was determined according to the second method of acid hydrolysis of GB 5009.6-2016. Protein was determined according to the first method of kjeldahl nitrogen determination of GB 5009.5-2016. Starch was determined according to the first method of enzyme hydrolysis of GB 5009.9-2023. Total sugar was determined according to GB/T 20977-2007. Dietary fiber was determined according to GB 5009.88-2023.

#### 2.3.2 Measurement of harmful factors

Trans-fatty acids were determined according to GB 5009.257-2016. Acrylamide was determined by liquid chromatography-mass spectrometry with dilution of stable isotopes according to GB 5009.204-2014, the first method. Polycyclic aromatic hydrocarbons (PAHs) were determined by gas chromatography–mass spectrometry according to GB 5009.265-2021, the first method.

### 2.4 GC-IMS detection methods

#### 2.4.1 Samples handling methods

The samples were placed in a 20 mL headspace vial (Aladdin Biochemical Technology Co., Ltd., Shanghai, China) and precisely weighed at 2 g. They were incubated at 60°C for 15 min. And then the samples were determined. Three sets of parallels were determined for each sample.

#### 2.4.2 GC-IMS measurements

The potato chip samples were analyzed using the GC-IMS method composed of Agilent gas chromatography (490 Agilent Technologies, Palo Alto, CA, United States) and IMS instrument (FlavourSpec®, Gesellschaft für Analytische Sensorsysteme mbH, Dortmund, Germany) and equipped with an automatic sampler (CTC-PAL 3 Analytics AG Company, Switzerland).

Headspace injection conditions: The incubation temperature was 80°C. The incubation time was 15 min. The injection volume was 500 μL. The sample was a non-split injection. The incubation speed was 500 r/min. The temperature of the injection needle was 85°C. At the same time, the sample was injected into the headspace.

GC conditions: The volatile flavor compounds were separated by a DB-WAX capillary chromatography column (15 m × 0.53 mm, 1.0 μm). The temperature of the column was 60°C. The carrier gas was high-purity helium (purity ≥99.999%). The programmed boosting was an initial flow rate of 2.00 mL/min held for 2 min, linearly increased to 10.00 mL/min within 8 min, and linearly increased to 100.00 mL/min within 10 min and held for 10 min. The chromatographic run time was 30 min. The injection port temperature was 80°C.

IMS conditions: Nitrogen (purity ≥99.999%) was used as the drift gas with a flow rate of 150 mL/min. The volatiles were ionized in the IMS ionization chamber (positive ion mode), and the ions were driven to a 53 mm migration tube at 45°C. The retention index (RI) of each volatile compound was calculated by the Laboratory Analytical Viewer (LAV) using n-ketones C4-C9 (Sinopharm Chemical Reagent Beijing Co., Ltd., Beijing, China) as external references. The volatile compounds were identified based on the retention index (RI) and drift time (RIP relative) of the standards in the GC-IMS Library. The Reporter plug-in and Gallery Plot plug-in were used to form the spectrogram and volatile fingerprints of samples (13).

### 2.5 Calculation of relative odor activity values

Relative odor activity value (ROAV) is a method that can more objectively and comprehensively assess the aroma contribution of a certain aroma component. It is considered based on the content of the aroma component and the aroma threshold. To determine the ROAV for each fragrance component, the OAV values of the other aroma components are compared to the OAV value of the aroma component with the highest OAV value, which serves as a criterion (14). The formula for ROAV is as follows:


$$\mathrm{ROAV}=\left(C/C_{\text{max}}\right)\times \left(T_{\text{max}}/T\right)\times 100.$$

Where T is the corresponding threshold value (μg/kg) for each volatile flavor compound. T_max_ is the threshold value (μg/kg) for the compound with the largest contribution. C is the relative content of each volatile flavor compound. And C_max_ is the relative content of the compound with the largest contribution.

### 2.6 Sensory evaluation

For the sensory evaluation of potato chips, 20 trained participants (20–40 years old) with a background in food science-related fields were chosen. 3-(methylsulfanyl)propanal solution (potato flavor), (E, E)-2,4-decadienal solution (deep-fried flavor), and (E)-2-nonenal solution (fat flavor) were used as references for flavor attributes of the potato chips (15). Before the start of the formal experiment, the panelists were informed of the objectives of the participation assessment, detailed experimental procedures, and sensory requirements. Five potato chips were analyzed from the six dimensions of potato flavor, deep-fried flavor, fatty flavor, greasiness, taste, and overall preference. Each sample was randomly coded and presented to the panelists in a colorless transparent bowl. Mouthwash with tasteless and odorless water when tasting different samples. The sensory evaluation table was shown in Table 1.

**Table 1:** Sensory evaluation table for potato chips.

Items	Scoring criteria and score
Potato flavor	Strong potato flavor (8–10)
	Slightly potato flavored, no off-flavors (4–7)
	No potato flavor or distinct odor (1–3)
Deep-fried flavor	Has a strong fried flavor (8–10)
	Slightly fried flavor, no off-flavors (4–7)
	No fried flavor or noticeable off-flavor (1–3)
Fatty flavor	Strong fatty flavor (8–10)
	Slightly fatty, no off-flavors (4–7)
	No fatty odor or distinct odor (1–3)
Greasiness	No excessive oil on exterior, low overall oil content, no greasy feeling (8–10)
	Average exterior oil, slightly greasy feeling, not severe (4–7)
	Exterior layer of oil with a heavy greasy feel (1–3)
Taste	Has the distinctive flavor of potato chips, with a mellow, mouth-watering taste (8–10)
	Mellower flavor, no other off-flavors (4–7)
	Poor taste, rancid grease or other off-flavors (1–3)
Overall preference	Better (8–10)
	General (4–7)
	Worse (1–3)

### 2.7 Statistical analysis

The GC-IMS data was processed by the Laboratory Analytical Viewer (LAV, G.A.S., Dortmund, Germany) using three plug-ins and the GC-IMS Library Search (NIST database and IMS database). The topographic plots and fingerprints of volatile compounds were established by plugins of Reporter and Gallery Plot (G.A.S., Dortmund, Germany). The relative content of each volatile compound was calculated using the peak area normalization method. Results were expressed as mean ± standard deviation (SD) and statistically analyzed using SPSS software (version 23.0, SPSS Inc., United States). Significance was analyzed using one-way analysis of variance (ANOVA) with Duncan’s *post hoc* test. A significance level of *p* < 0.05 was considered statistically significant. Origin 2018 (OriginLab Corporation, Northampton, MA, United States) was used for plotting. To ensure reliability, all experimental results were measured three times concurrently at least.

## 3 Results and discussion

### 3.1 Physicochemical properties

#### 3.1.1 The fundamental ingredients of different potato chips

Table 2 displayed the findings of the analysis of the fundamental ingredients of the five distinct varieties of potato chips. Both LS industrial fresh-cut fried potato chips and KBK industrial composite fried potato chips had fat contents as high as 32.36 and 30.33%, respectively. In comparison to the fried potato chips, the fat content of the SYS, SY, and XSC composite baked potato chips was significantly lower, ranging from 21.29 to 22.21% (*p* < 0.05). Studies have shown that fried potato chips absorb a relatively large amount of oil, usually more than 30% (16). This is consistent with the result of the study. When compared to the frying process, the baking process was able to drastically lower the fat level of potato chips. It was mainly due to the limitation of the process that it was difficult to control the fat content of potato chips in the deep-frying process. Despite numerous studies on controlling slice thickness, deep-frying temperature, and time, the oil content of fried potato chips remained high. The fat content of potato chips can be artificially and successfully controlled during the baking process, and there was less variation in the fat content of composite baked potato chips made using various formulations.

**Table 2:** Basic components of potato chips.

Items	LS	KBK	SYS	SY	XSC
Fat%	32.36 ± 0.28^a^	30.33 ± 0.32^b^	22.21 ± 0.02^c^	21.29 ± 0.34^d^	21.77 ± 0.08^cd^
Protein%	7.83 ± 0.08^a^	5.27 ± 0.02^b^	5.00 ± 0.06^c^	5.27 ± 0.02^b^	3.95 ± 0.01^d^
Starch%	44.94 ± 0.98^c^	48.18 ± 0.64^b^	43.79 ± 0.86^c^	52.07 ± 1.07^a^	50.88 ± 1.80^a^
Dietary fiber%	4.40 ± 0.14^c^	4.41 ± 0.17^c^	9.56 ± 0.37^a^	5.08 ± 0.52^b^	3.95 ± 0.15^c^

Means superscripted by different letters in the same row are significantly different at *p* < 0.05.

The protein level of potato chips varied significantly (*p* < 0.05). LS industrial fresh-cut fried potato chips had the greatest protein amount, at 7.83%. The Maillard reaction between starch and protein during potato chip processing results in a number of flavor compounds that help to maintain the chips’ flavor and aroma (17). Furthermore, the starch percentage of the five potato chips varied between 43.19 and 52.07%, which was the highest of the ingredients and gave the chips their crispy quality. The SYS homemade composite baked potato chips prepared in this study were high-dietary fiber potato chips with dietary fiber content up to 9.56%, which had the effect of promoting digestive health and controlling blood glucose and blood lipids.

#### 3.1.2 Harmful factors of different potato chips

Table 3 displayed the findings of the analysis of the harmful factors of five distinct potato chips. During food preparation, a number of intricate chemical reactions will take place in the food components to create particular sensory qualities, but these reactions will also result in the production of harmful factors such as acrylamide, trans-fatty acids, and polycyclic aromatic hydrocarbons (PAHs) (18). Table 3 showed that none of the five potato chips contained trans-fatty acids. All five potato chips contained acrylamide, although the greatest concentration was found in LS industrial fresh-cut fried potato chips (399.10 μg/kg), followed by KBK industrial composite fried potato chips (393.35 μg/kg). These two fried potato chips had a substantially greater acrylamide concentration than the composite baked potato chips (104.91–164.41 μg/kg). The acrylamide content in potato chips in the new 2018 EU Act was limited to 750 μg/kg. The acrylamide content of five potato chips in this study was within the limited range. The previous research had shown that the acrylamide content of baked potato chips was lower than that of fried potato chips under 180°C and 190°C processing conditions, which was consistent with the results of the present study (19). In terms of acrylamide, consumers are more willing to choose baked potato chips. Acrylamide can be produced by the direct breakdown of asparagine-Amadori compounds or by the reactivity of *α*-dicarbonyl compounds with asparagine in the Maillard reaction. And potatoes contain the higher amount of asparagine, which readily forms acrylamide (20).

**Table 3:** Harmful factors of potato chips.

Items		LS	KBK	SYS	SY	XSC
Trans-fatty acids (%)		–	–	–	–	–
Acrylamide (μg/kg)		399.10 ± 10.26^a^	393.35 ± 9.00^b^	164.41 ± 4.41^c^	104.91 ± 2.68^d^	155.93 ± 6.96^c^
Polycyclic aromatic hydrocarbons (μg/kg)	Dibenzo[a,e]pyrene	7.80 ± 0.18^a^	6.14 ± 0.89^a^	2.81 ± 0.20^b^	6.15 ± 1.84^a^	1.62 ± 1.65^b^
	Dibenzo[a,i]pyrene	11.69 ± 0.22^a^	11.11 ± 0.55^a^	4.06 ± 0.32^c^	7.14 ± 1.43^b^	5.36 ± 0.54^bc^
	Dibenzo[a,h]pyrene	10.26 ± 4.74^a^	9.52 ± 0.03^ab^	3.72 ± 0.38^c^	6.88 ± 2.18^ab^	6.64 ± 0.05^ab^
	Dibenzo[a,l]pyrene	7.27 ± 0.06^a^	4.68 ± 0.90^b^	1.35 ± 0.09^c^	–	–
	Benzo[c]fluorene	–	1.92 ± 0.14^a^	–	–	0.71 ± 0.01^b^
	Indeno[1,2,3-c,d]pyrene	–	–	0.19 ± 0.01	–	–
	Benzo[a]anthracene	–	–	–	–	–
	Cyclopenta[c,d]pyrene	–	–	–	–	–
	Chrysene	–	–	–	–	–
	5-Methylchrysene	–	–	–	–	–
	Benzo[b]fluoranthene	–	–	–	–	–
	Benzo[k]fluoranthene	–	–	–	–	–
	Benzo[j]fluoranthene	–	–	–	–	–
	Benzo[a]pyrene	–	–	–	–	–
	Dibenzo[a,h]anthracene	–	–	–	–	–
	Benzo[g,h,i]perylene	–	–	–	–	–
	Total PAHs	37.02	33.37	12.13	20.17	14.33

Means superscripted by different letters in the same row are significantly different at *p* < 0.05.

This investigation found just six of the 16 PAHs, including indeno[1,2,3-c,d]pyrene, benzo[c]fluorene, dibenzo[a,e]pyrene, dibenzo[a,i]pyrene, dibenzo[a,h]pyrene, and dibenzo[a,l]pyrene. Although there is currently no established limit for other PAHs, the GB 2762-2017 national standard for food safety limits for contaminants in foods sets the benzo[a]pyrene (BaP) limit at 5 μg/kg. BaP was not found in the five potato chips used in this study. The high fat and protein content of fried potato chips is a significant factor influencing the formation of PAHs. It may be the reason why the total PAHs in KBK industrial composite fried potato chips and LS industrial fresh-cut fried potato chips were higher than those in composite baked potato chips (21, 22). In conclusion, fried potato chips have lower harmful factors than baked potato chips.

### 3.2 Spectral analysis of volatile flavor compounds in different potato chips

**Figure 1:**
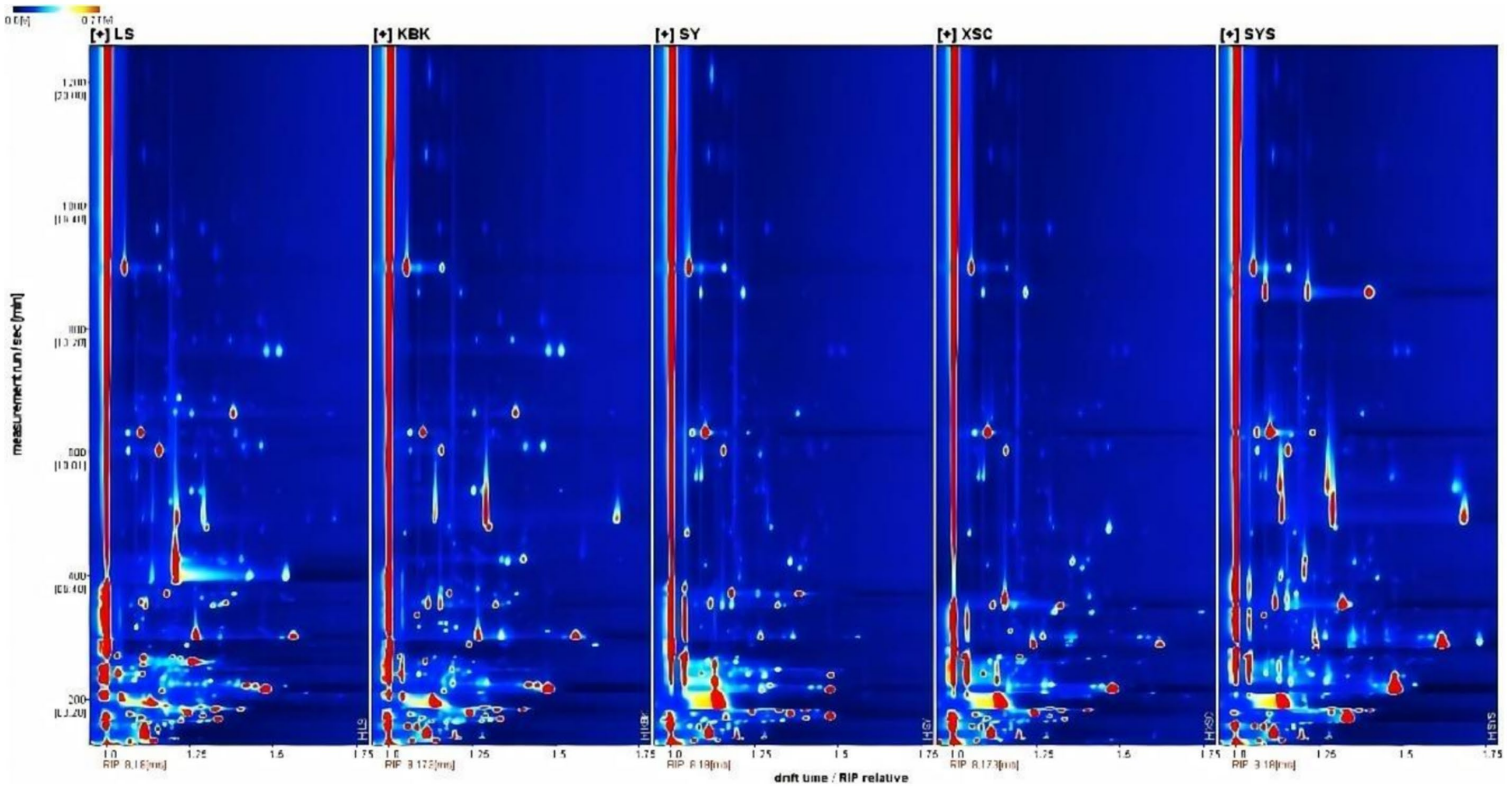
Two-dimensional GC-IMS spectrum of the potato chips.

**Figure 2:**
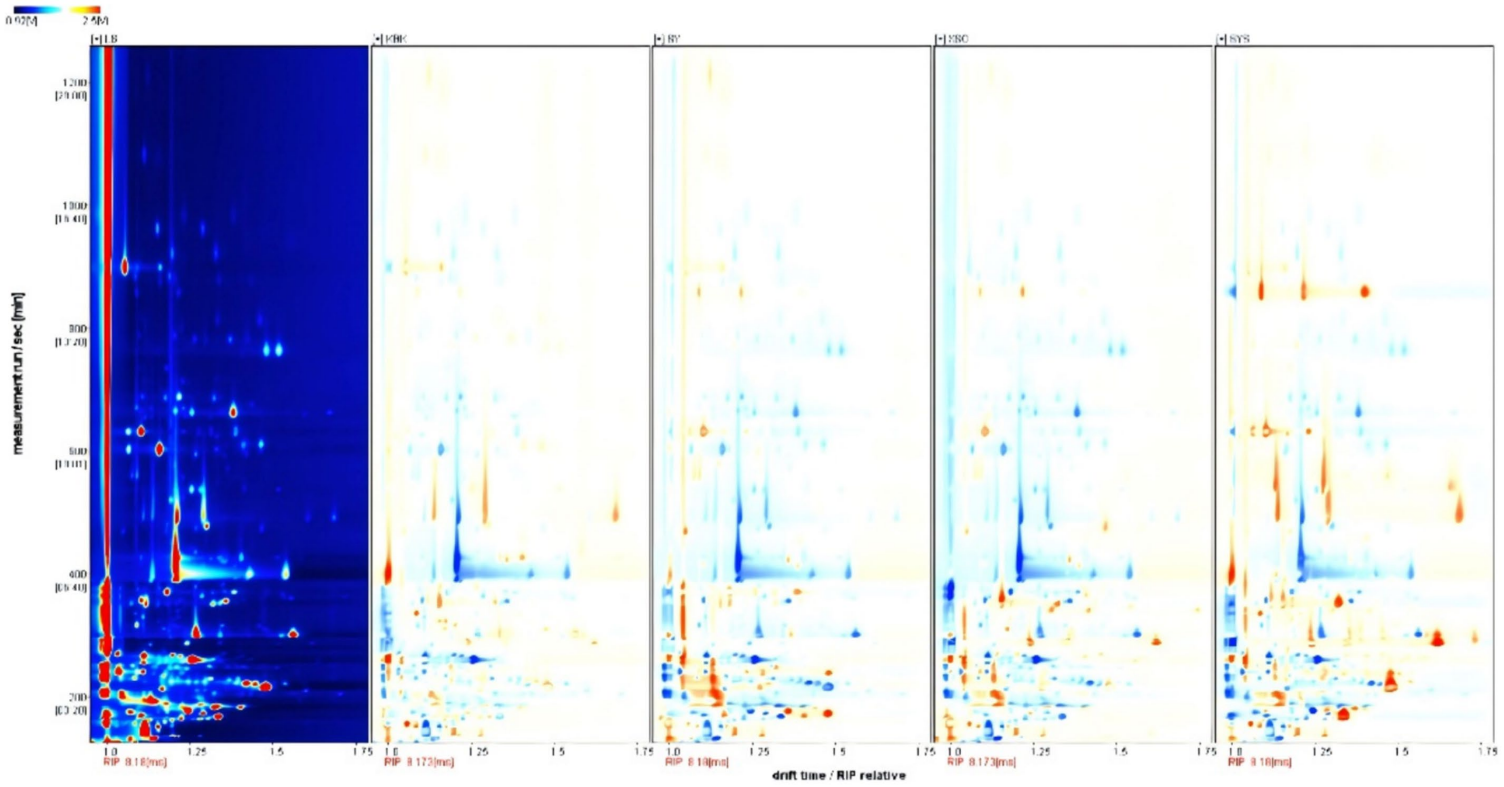
GC-IMS spectrum of the potato chips (difference diagram). 1. The background of the spectrum is blue; the vertical coordinate indicates the GC retention time, and the horizontal coordinate indicates the relative ion mobility time. 2. The color represents the concentration of the substance; white indicates a low concentration, and red indicates a high concentration; the darker the color, the higher the concentration.

A food’s flavor is its distinct impression as experienced by the senses of taste and smell. The volatile compounds’ composition and concentration in potato chips made using various formulations and processing techniques vary somewhat. Due to the differences in concentration and nature, some volatile flavor compounds were detected not only as monomers (-M) but also as dimers (-D). LS industrial fresh-cut fried potato chips, KBK industrial composite fried potato chips, SY industrial composite baked potato chips, XSC industrial composite baked potato chips, and SYS homemade composite baked potato chips were displayed from left to right in Figures 1, 2. In Figure 2, the darker the color, the larger the difference. The blue area showed that the compound was lower than LS, while the red area showed that the compound was higher than LS. The volatile flavor compounds of KBK industrial composite fried potato chips and SYS homemade composite baked potato chips differed significantly from those of other samples.

### 3.3 Qualitative analysis of volatile flavor compounds in different potato chips

A total of 57 volatile flavor compounds (including monomer-M and dimer-D) were detected by the GC-IMS technique in Table 4, including 17 aldehydes (25.33–33.88%), 9 esters (15.52–19.60%), 10 ketones (12.40–22.45%), 3 pyrazines (2.33–7.38%), 12 alcohols (16.27–23.98%), 3 acids (3.78–10.79%), 1 pyridine (1.01–3.32%), and 2 ethers (0.83–3.77%). Among these detected compounds, aldehydes had the highest type and relative content. It indicated that aldehydes played a major role in the presentation of the flavor of different types of potato chips, followed by esters, ketones, and alcohols.

**Table 4:** Classification of volatile flavor compounds in potato chips.

Compounds classification	Type	LS	KBK	SYS	SY	XSC
Aldehydes	17	25.33 ± 3.91^a^	30.32 ± 3.49^a^	33.80 ± 1.67^a^	33.88 ± 7.64^a^	28.70 ± 2.95^a^
Esters	9	19.60 ± 6.65^a^	15.64 ± 3.54^a^	15.52 ± 0.45^a^	15.57 ± 4.52^a^	15.64 ± 6.17^a^
Ketones	10	18.74 ± 2.32^ab^	16.53 ± 3.26^ab^	19.80 ± 2.69^ab^	12.40 ± 2.26^b^	22.45 ± 7.07^a^
Pyrazines	3	7.38 ± 5.10^a^	5.08 ± 3.06^a^	5.46 ± 1.31a	5.52 ± 2.24^a^	2.33 ± 1.70^a^
Alcohols	12	17.63 ± 8.91^a^	18.62 ± 2.30^a^	16.27 ± 1.08a	19.72 ± 3.09^a^	23.98 ± 3.99^a^
Acids	3	6.54 ± 2.41^ab^	10.79 ± 0.39^a^	5.84 ± 2.30b	6.94 ± 2.14^b^	3.78 ± 2.15^b^
Pyridines	1	1.01 ± 1.56^a^	1.05 ± 0.13^a^	2.48 ± 0.08a	3.32 ± 2.46^a^	1.41 ± 1.77^a^
Ethers	2	3.77 ± 1.77^a^	1.97 ± 0.08^ab^	0.83 ± 0.87b	2.67 ± 1.90^ab^	1.71 ± 1.01^ab^

Means superscripted by different letters in the same row are significantly different at *p* < 0.05.

### 3.4 Fingerprints and relative contents of volatile flavor compounds in different potato chips

**Figure 3:**
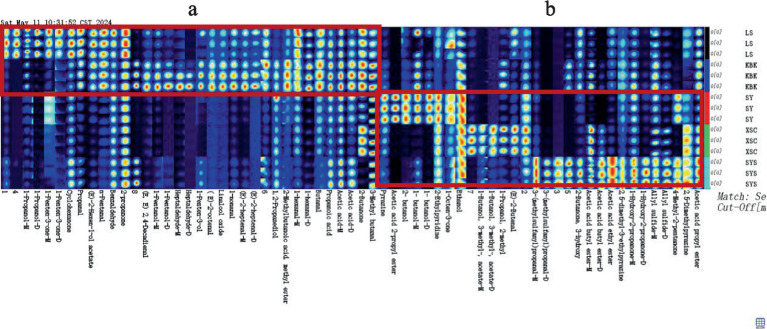
Gallery plot diagram of volatile flavor compounds in the potato chips. Note: Each row represents all the signal peaks selected in the sample, and each column represents the difference of signal peaks of the same substance in different samples; the darker the color, the higher the concentration of the compound and the stronger the signal peaks. “-M” and “-D” denote monomer and dimer, respectively. The zone “a” showed the variations in volatile flavor compounds between various fried potato chips. The zone “b” displayed the variations in volatile flavor compounds between various composite baked potato chips.

In Figure 3, the fingerprints can be relatively clear to compare the variability of volatile flavor compounds of five different potato chips (23). The zone “a” showed that fried potato chips were richer in volatile flavor compounds, and there was a significant difference in volatile flavor substances between LS industrial fresh-cut fried potato chips and KBK industrial composite fried potato chips that can be observed. However, because different composite baked potato chip formulations and processes differ, zone “b” displayed the variations in volatile flavor compounds between various composite baked potato chips.

Table 5 presented the mean and standard deviation of the relative content of each volatile compound and explored the differences in volatile flavor compounds among the different potato chips. Most of the aldehydes originate from the oxidation of unsaturated fatty acids by oxidation (24). The oxidation of unsaturated fatty acids during high-temperature frying and baking of potato chips can produce hydroperoxides, which can then be pyrolyzed to produce aliphatic aldehydes (25). 3-(methylsulfanyl)propanal (-M and -D) has a potato aroma (15). Moreover, LS industrial fresh-cut fried potato chips had the highest content (5.11%), followed by SYS homemade composite baked potato chips and SY industrial composite baked potato chips (4.61 and 4.57%, respectively). This was consistent with the results of the sensory evaluation of potato flavor in potato chips. 1-nonanal, (E)-2-heptenal (-M and -D), heptaldehyde (-M and -D), and 1-hexanal (-M and -D) contributed the fatty flavor to the potato chips. (E, E)-2,4-decadienal was mainly produced by the oxidation of the unsaturated fatty acid linoleic acid, which was able to add deep-fried flavor to potato chips (26). The present study showed that there was a significant difference in the relative content of (E, E)-2,4-decadienal between fried and baked potato chips. The content of this compound was 2.55 and 3.52% in LS industrial fresh-cut fried potato chips and KBK industrial composite fried potato chips, respectively. However, it was lower in baked potato chips. This also led to the rich deep-fried flavor of fried potato chips.

**Table 5:** Relative contents of the volatile flavor compounds of potato chips.

Count	Compound name	CAS	Formula	MW	RI	Rt [sec]	Dt [a.u.]	Relative content (%)				
								LS	KBK	SYS	SY	XSC
Aldehydes (17)												
1	(E, E)2,4-Decadienal	C25152845	C10H16O	152.2	1310.9	61,073	141,449	2.55 ± 0.71^ab^	3.52 ± 0.72^a^	0.67 ± 0.50^b^	0.86 ± 0.65^ab^	1.30 ± 0.22^ab^
2	(E)-2-Octenal	C2548870	C8H14O	126.2	1434.8	815,669	133,586	1.50 ± 0.35^a^	1.85 ± 0.45^a^	0.57 ± 0.44^a^	3.12 ± 0.69^a^	2.16 ± 0.08^a^
3	Benzaldehyde	C100527	C7H6O	106.1	1507.5	966,498	105,443	2.04 ± 0.41^a^	1.82 ± 0.39^a^	2.68 ± 0.10^a^	1.11 ± 0.65^a^	0.89 ± 0.50^a^
4	3-(Methylsulfanyl)propanal-M	C3268493	C4H8OS	104.2	1457.4	85,981	109,136	1.53 ± 0.13^ab^	2.25 ± 0.07^ab^	1.95 ± 0.06^ab^	3.32 ± 1.90^a^	0.45 ± 0.07^b^
5	3-(Methylsulfanyl)propanal-D	C3268493	C4H8OS	104.2	1457.2	859,533	140,182	3.58 ± 0.36^a^	0.91 ± 0.28^a^	2.62 ± 0.27^a^	1.29 ± 0.29^a^	0.91 ± 0.71^a^
6	1-Nonanal	C124196	C9H18O	142.2	1407.9	766,051	148,275	1.63 ± 0.75^a^	0.67 ± 0.07^a^	2.52 ± 0.04^a^	1.09 ± 0.79^a^	0.78 ± 0.53^a^
7	(E)-2-Heptenal-M	C18829555	C7H12O	112.2	1347.7	665,466	125,498	0.79 ± 0.07^a^	2.59 ± 0.03^a^	3.07 ± 0.90^a^	0.75 ± 0.15^a^	2.89 ± 0.06^a^
8	(E)-2-Heptenal-D	C18829555	C7H12O	112.2	1346.9	664,234	167,262	1.03 ± 0.44^a^	0.77 ± 0.07^a^	1.49 ± 0.56^a^	3.25 ± 0.51^a^	1.78 ± 0.01^a^
9	Heptaldehyde-M	C111717	C7H14O	114.2	1197.5	426,787	133,784	1.62 ± 0.19^a^	0.67 ± 0.31^a^	1.01 ± 0.05^a^	1.26 ± 0.11^a^	0.60 ± 0.05^a^
10	Heptaldehyde-D	C111717	C7H14O	114.2	1198.6	428,386	169,429	0.73 ± 0.18^b^	4.82 ± 1.81^a^	0.45 ± 0.11^b^	1.96 ± 0.34^ab^	1.67 ± 0.32^ab^
11	(E)-2-Butenal	C123739	C4H6O	70.1	1064.8	270,771	12,002	0.89 ± 0.46^a^	0.72 ± 0.53^a^	1.38 ± 0.09^a^	1.14 ± 0.86^a^	0.06 ± 0.02^a^
12	n-Pentanal	C110623	C5H10O	86.1	1004.0	224,827	141,987	1.15 ± 0.18^b^	0.82 ± 0.04^b^	1.19 ± 0.86^b^	1.87 ± 0.37^ab^	2.91 ± 0.48^a^
13	3-Methyl butanal	C590863	C5H10O	86.1	932.1	184,726	140,235	0.57 ± 0.07^a^	3.88 ± 0.90^a^	2.06 ± 0.80^a^	3.24 ± 0.40^a^	3.33 ± 0.36^a^
14	Butanal	C123728	C4H8O	72.1	891.2	165,473	127,938	1.45 ± 0.04^bc^	2.54 ± 0.08^b^	7.61 ± 0.72^a^	1.54 ± 0.09^bc^	1.27 ± 0.23^c^
15	Propanal	C123386	C3H6O	58.1	815.2	134,861	114,284	1.24 ± 0.98^a^	0.97 ± 0.05^a^	0.59 ± 0.02^a^	2.84 ± 0.42^a^	2.60 ± 0.57^a^
16	1-Hexanal-M	C66251	C6H12O	100.2	1101.1	303,149	127,104	1.55 ± 0.29^a^	0.74 ± 0.55^a^	2.26 ± 0.67^a^	2.72 ± 0.56^a^	2.65 ± 0.72^a^
17	1-Hexanal-D	C66251	C6H12O	100.2	1100.8	302,832	156,329	1.46 ± 0.35^a^	0.80 ± 0.06^a^	1.66 ± 0.21^a^	2.53 ± 0.10^a^	2.44 ± 0.93^a^
Acids (3)												
18	Acetic acid-M	C64197	C2H4O2	60.1	1478.8	903,993	105,443	3.00 ± 0.25^a^	0.22 ± 0.19^b^	2.18 ± 0.61^ab^	2.37 ± 0.80^ab^	1.04 ± 0.49^ab^
19	Acetic acid-D	C64197	C2H4O2	60.1	1477.1	900,455	116,449	1.83 ± 0.55^b^	6.04 ± 0.24^a^	0.48 ± 0.04^b^	1.75 ± 0.20^b^	1.58 ± 0.39^b^
20	Propanoic acid	C79094	C3H6O2	74.1	1557.2	1085.61	111,758	1.70 ± 0.19^a^	4.53 ± 0.42^a^	3.18 ± 0.42^a^	2.81 ± 0.04^a^	1.16 ± 0.46^a^
Alcohols (12)												
21	1,2-Propanediol	C57556	C3H8O2	76.1	1605.6	1,215,337	112,488	0.25 ± 0.09^a^	1.84 ± 0.35^a^	1.88 ± 0.53^a^	1.60 ± 0.60^a^	3.11 ± 0.37^a^
22	Linalool oxide	C60047178	C10H18O2	170.3	1418.3	784,906	12,638	0.94 ± 0.79^b^	2.01 ± 0.22^ab^	0.58 ± 0.12^b^	0.76 ± 0.21^b^	5.20 ± 0.43^a^
23	1-Pentanol-M	C71410	C5H12O	88.1	1269.0	540,177	125,504	1.18 ± 0.30^a^	0.72 ± 0.11^a^	1.71 ± 0.22^a^	1.42 ± 0.24^a^	1.03 ± 0.14^a^
24	1-Pentanol-D	C71410	C5H12O	88.1	1268.3	539,005	151,824	0.72 ± 0.19^a^	1.17 ± 0.06^a^	2.27 ± 0.92^a^	2.91 ± 0.07^a^	1.22 ± 0.47^a^
25	1-Propanol-M	C71238	C3H8O	60.1	1054.6	262,442	11,139	1.48 ± 0.13^ab^	0.53 ± 0.40^b^	2.02 ± 0.19^ab^	0.95 ± 0.71^ab^	4.84 ± 0.36^a^
26	1-Propanol-D	C71238	C3H8O	60.1	1052.9	261,099	126,633	1.56 ± 0.16^a^	1.95 ± 0.45^a^	1.22 ± 0.58^a^	1.96 ± 0.43^a^	1.13 ± 0.58^a^
27	Ethanol	C64175	C2H6O	46.1	952.2	195,004	114,416	2.55 ± 0.39^a^	2.08 ± 0.55^a^	2.29 ± 0.73^a^	2.72 ± 0.32^a^	1.29 ± 0.83^a^
28	1-Propanol, 2-methyl	C78831	C4H10O	74.1	1110.0	313,001	117,191	0.69 ± 0.21^a^	1.19 ± 0.87^a^	0.76 ± 0.08^a^	3.16 ± 0.92^a^	0.32 ± 0.09^a^
29	1-Penten-3-ol	C616251	C5H10O	86.1	1174.5	393,721	94,017	2.06 ± 0.94^a^	2.04 ± 0.53^a^	0.92 ± 0.69^a^	0.93 ± 0.73^a^	0.79 ± 0.34^a^
30	1-Butanol-M	C71363	C4H10O	74.1	1158.6	372,111	118,479	2.26 ± 0.39^a^	2.79 ± 0.55^a^	0.42 ± 0.03^a^	1.33 ± 0.12^a^	1.26 ± 0.30^a^
31	1-Butanol-D	C71363	C4H10O	74.1	1158.0	371,371	138,713	0.23 ± 0.14^a^	0.72 ± 0.89^a^	1.40 ± 0.69^a^	0.91 ± 0.08^a^	2.44 ± 0.72^a^
32	2-Butanol	C78922	C4H10O	74.1	1037.8	249,274	132,168	3.71 ± 0.35^a^	1.58 ± 0.32^a^	0.78 ± 0.95^a^	1.07 ± 0.59^a^	1.34 ± 0.29^a^
Pyrazines (3)												
33	2,5-Dimethyl-3-ethylpyrazine	C13360651	C8H12N2	136.2	1476.9	899.98	109,342	1.18 ± 0.79^a^	2.26 ± 0.93^a^	3.59 ± 0.72^a^	2.16 ± 0.55^a^	1.06 ± 0.51^a^
34	Pyrazine	C290379	C4H4N2	80.1	1227.2	470,703	105,074	2.95 ± 0.38^a^	1.34 ± 0.97^a^	1.08 ± 0.50^a^	2.84 ± 0.71^a^	0.31 ± 0.06^a^
35	2,5-Dimethylpyrazine	C123320	C6H8N2	108.1	1342.1	656.92	111,855	3.25 ± 0.38^a^	1.48 ± 0.38^ab^	0.80 ± 0.07^b^	0.51 ± 0.07^b^	0.96 ± 0.14^b^
Ketones (10)												
36	1-Hydroxy-2-propanone-M	C116096	C3H6O2	74.1	1326.5	633,437	106,375	2.50 ± 0.76^a^	2.92 ± 0.50^a^	0.62 ± 0.46^a^	0.58 ± 0.08^a^	0.41 ± 0.08^a^
37	1-Hydroxy-2-propanone-D	C116096	C3H6O2	74.1	1327.2	634,361	12,305	0.59 ± 0.04^a^	2.46 ± 0.21^a^	4.16 ± 0.05^a^	1.14 ± 0.40^a^	1.82 ± 0.09^a^
38	2-Butanone, 3-Hydroxy	C513860	C4H8O2	88.1	1306.6	604,577	10,687	3.83 ± 0.91^a^	1.97 ± 0.54^a^	3.35 ± 0.49^a^	0.77 ± 0.05^a^	1.21 ± 0.12^a^
39	1-Octen-3-one	C4312996	C8H14O	126.2	1301.8	597,905	12,705	0.68 ± 0.21^ab^	1.43 ± 0.09^ab^	0.43 ± 0.03^b^	0.31 ± 0.09^b^	2.36 ± 0.59^a^
40	1-Penten-3-one-M	C1629589	C5H8O	84.1	1014.4	232,081	108,364	2.06 ± 0.62^a^	2.18 ± 0.80^a^	3.02 ± 0.19^a^	1.32 ± 0.03^a^	3.06 ± 0.40^a^
41	1-Penten-3-one-D	C1629589	C5H8O	84.1	1013.6	231,544	131,116	1.53 ± 0.15^b^	0.58 ± 0.07^b^	0.29 ± 0.08^b^	0.38 ± 0.33^b^	4.40 ± 1.02^a^
42	4-Methyl-2-pentanone	C108101	C6H12O	100.2	1025.6	240,142	148,152	3.58 ± 0.70^a^	0.33 ± 0.25^a^	2.96 ± 0.88^a^	0.48 ± 0.35^a^	3.71 ± 0.65^a^
43	2-Butanone	C78933	C4H8O	72.1	921.4	179,463	124,619	1.50 ± 0.19^a^	1.93 ± 0.12^a^	1.16 ± 0.86^a^	0.89 ± 0.04^a^	1.63 ± 0.29^a^
44	2-Propanone	C67641	C3H6O	58.1	842.8	145.25	111,191	0.81 ± 0.11^b^	1.92 ± 0.56^ab^	2.23 ± 0.85^ab^	5.76 ± 0.28^a^	3.53 ± 0.81^ab^
45	Cyclohexanone	C108941	C6H10O	98.1	1306.1	603,877	115,926	1.66 ± 0.23^a^	0.81 ± 0.14^a^	1.60 ± 0.13^a^	0.77 ± 0.57^a^	0.31 ± 0.03^a^
Esters (9)												
46	(E)-2-Hexen-1-ol acetate	C2497189	C8H14O2	142.2	1346.3	663.31	138,196	1.05 ± 0.82^a^	0.93 ± 0.17^a^	1.73 ± 0.48^a^	2.44 ± 0.80^a^	1.74 ± 0.49^a^
47	Acetic acid propyl ester	C109604	C5H10O2	102.1	991.6	216,767	148,264	2.89 ± 0.29^a^	0.30 ± 0.02^a^	0.84 ± 0.04^a^	0.82 ± 0.08^a^	3.03 ± 0.13^a^
48	2-Methylbutanoic acid, methyl ester	C868575	C6H12O2	116.2	1038.1	249,545	119,012	1.56 ± 0.22^a^	2.43 ± 0.81^a^	0.60 ± 0.14^a^	1.16 ± 0.85^a^	0.83 ± 0.59^a^
49	Acetic acid ethyl ester	C141786	C4H8O2	88.1	900.7	169,767	133,671	0.92 ± 0.06^a^	0.61 ± 0.02^a^	1.70 ± 0.17^a^	3.34 ± 0.20^a^	1.73 ± 0.18^a^
50	Acetic acid 2-propyl ester	C108214	C5H10O2	102.1	910.6	174,338	14,808	2.24 ± 0.72^a^	2.63 ± 0.80^a^	3.84 ± 0.80^a^	1.58 ± 0.17^a^	3.94 ± 0.82^a^
51	Acetic acid butyl ester-M	C123864	C6H12O2	116.2	1086.3	289,166	124,401	1.67 ± 0.14^a^	0.85 ± 0.05^a^	0.98 ± 0.73^a^	1.18 ± 0.92^a^	1.48 ± 0.34^a^
52	Acetic acid butyl ester-D	C123864	C6H12O2	116.2	1086.6	289,484	162,122	3.62 ± 0.06^a^	3.17 ± 0.15^a^	2.50 ± 0.83^a^	3.58 ± 0.82^a^	0.77 ± 0.53^a^
53	1-Butanol, 3-methyl-, acetate-M	C123922	C7H14O2	130.2	1135.6	342,847	130,222	2.96 ± 0.29^a^	2.19 ± 0.69^a^	1.64 ± 0.24^a^	0.40 ± 0.02^a^	0.35 ± 0.29^a^
54	1-Butanol, 3-methyl-, acetate-D	C123922	C7H14O2	130.2	1135.6	342,847	174,492	2.67 ± 0.00^a^	2.53 ± 0.58^a^	1.70 ± 0.14^a^	1.06 ± 0.32^a^	1.74 ± 0.46^a^
Pyridines (1)												
55	2-Ethylpyridine	C100710	C7H9N	107.2	1281.7	563,305	109,242	1.01 ± 0.56^a^	1.05 ± 0.13^a^	2.48 ± 0.08^a^	3.32 ± 0.46^a^	1.41 ± 0.77^a^
Ethers (2)												
56	Allyl sulfide-M	C592881	C6H10S	114.2	1145.1	354,632	11,217	1.02 ± 0.25^a^	1.18 ± 0.03^a^	0.61 ± 0.06^a^	0.92 ± 0.13^a^	0.83 ± 0.24^a^
57	Allyl sulfide-D	C592881	C6H10S	114.2	1145.1	354,632	132,125	2.75 ± 0.98^a^	0.79 ± 0.06^a^	0.22 ± 0.09^a^	1.75 ± 1.00^a^	0.88 ± 0.08^a^

Means superscripted by different letters in the same row are significantly different at *p* < 0.05. The compound suffixes M and D are the monomer and dimer of the same compound, respectively. Formula, molecular formula of the compound; MW, molecular weight; Ri, retention index; Rt, retention time (sec); Dt, Relative migration time, migration time / RIP migration time, dimensionless (a.u.).

Esters, which give potato chips a pleasant flavor, are mainly produced when fats oxidize or when alcohols combine with carboxylic acid compounds in potatoes. The esters detected in this study, such as (E)-2-hexen-1-ol acetate, acetic acid propyl ester, acetic acid ethyl ester, acetic acid 2-propyl ester, 1-butanol-3-methyl-acetate (-D and -M), acetic acid butyl ester (-D and -M), and 2-methylbutanoic acid methyl ester, were able to impart a pleasant fruity aroma to the product. The highest total ester content of 19.60% was found in LS industrial fresh-cut fried potato chips, while the other composite baked potato chips had a total content of 15.52–15.64%. LS industrial fresh-cut fried chips had the highest content of acetic acid butyl ester (-D and -M) and 1-butanol-3-methyl-acetate (-D and -M) at 5.29 and 5.63%, respectively. SY industrial composite baked chips had the highest content of (E)-2-hexen-1-ol acetate and acetic acid ethyl ester at 2.44 and 3.34%, respectively. XSC industrial composite baked chips had the highest content of acetic acid propyl ester and acetic acid 2-propyl ester, at 3.03 and 3.94%, respectively.

Most of the ketones are products of the Maillard reaction and oxidative degradation of unsaturated fatty acids under the influence of heat (27). 2-butanone-3-hydroxy and 2-butanone are important compounds in imparting flavor to butter and cream (28, 29). The highest content of 2-butanone, 3-hydroxy was 3.83% in LS industrial fresh-cut fried potato chips, which was mainly due to the high volatile flavor compound content of this compound in raw potato tubers (30). 2-Propanone, a product of glucose catabolism, was found in high levels in SY industrial composite baked potato chips and may have originated from the use of potato powder made from some defective potatoes (31). 1-hydroxy-2-propanone (-M and -D) gives a caramel flavor to potato chips, and 2-butanone may originate from the breakdown of fats. These compounds were highest in KBK industrial composite fried potato chips. In XSC industrial composite baked potato chips, the highest relative levels were found in 1-octen-3-one, 1-penten-3-one (-M and -D), and 4-methyl-2-pentanone. Furthermore, the maximum relative level of earthy-flavored cyclohexanone was found in LS industrial fresh-cut fried potato chips.

Alcohol compounds are mainly produced through oxidative decomposition and reduction reactions of fats (32). Most of the alcohol compounds have an alcoholic flavor, such as 1,2-propanediol, 1-propanol, ethanol, 1-propanol-2-methyl, and 1-butanol in Table 5. 1-penten-3-ol contributes fruity flavor. Most of these alcohols originate from enzymatic oxidation by lipoxygenase. The contents of alcohol in fried potato chips, such as LS industrial fresh-cut fried potato chips and KBK industrial composite fried potato chips, were lower, at 17.63 and 18.62%, respectively. This may be due to the fact that high-temperature frying accelerates the further oxidation of alcohols to produce aldehydes and ketones.

Pyrazine compounds are mainly produced in the final stage of the Maillard reaction (33). Pyrazines are the most important compounds in potato flavor compounds with a strongly pleasant odor, such as fresh or baked potato flavor. The highest content of pyrazines was 7.38% in LS industrial fresh-cut fried potato chips, followed by SY industrial composite baked potato chips and SYS homemade composite baked potato chips. SXC industrial composite baked potato chips had 2.33% pyrazines, and their potato flavor was mild. Fresh-cut potato chips had higher levels of pyrazines than composite potato chips, which might be because the cooking procedure used to make potato flour destroys pyrazine volatile flavor components (14). However, the preparation process of composite baked potato chips can increase the content of pyrazine compounds by adding the flavor and spices, so as to improve the potato flavor of potato chips.

Among the other flavor compounds, only one pyridine substance, 2-ethylpyridine, was detected in the potato chips. This compound was found at a high level of 3.32% in SY industrial composite baked potato chips, while the lowest level was 1.01% in LS industrial fresh-cut fried potato chips. The ether compounds were mainly derived from the thermal degradation of amino acids or from the products of the Maillard reaction. And only diallyl sulfide (-D and -M) was detected in the potato chips at present. The highest level of this compound was 3.77% in LS industrial fresh-cut fried potato chips, while the lowest level was 0.83% in SYS composite baked potato chips.

### 3.5 Analysis of key volatile flavor compounds ROAV for different potato chips

A variety of volatile flavor compounds were detected in different potato chips, only some of which contributed significantly to the overall flavor of the potato chips. And the rest of the compounds mainly acted as modifiers and synergists to the overall flavor of the potato chips. The relative odor activity value (ROAV) is widely used to characterize the contribution of volatile flavor compounds to overall flavor (34). Compounds with ROAV ≥1 contribute more to the aroma of the sample and are key volatile flavor compounds. Additionally, the higher a compound’s ROAV value, the more substantial its contribution to the sample’s overall flavor. It is well acknowledged that compounds with 0.1 ≤ ROAV ≤1 are significant modifiers of the potato chip scent, while compounds with ROAV <0.1 contribute less to the sample flavor (35).

Table 6 showed the key volatile flavor compounds with ROAV ≥1. For the five potato chips, the same key volatile compounds with ROAV ≥1 were (E, E)2,4-decadienal, 3-(methylsulfanyl)propanal-D, 3-(methylsulfanyl)propanal-M, (E)-2-butenal, 1-hydroxy-2-propanone-D, 1-hydroxy-2-propanone-M, and 1-octen-3-one. The flavor compounds of LS industrial fresh-cut fried potato chips were more abundant, with 1-nonanal, butanal, 1-penten-3-one-D, 1-penten-3-one-M, acetic acid ethyl ester, and 1-butanol-3-methyl-acetate-M having ROAV ≥1, whereas these compounds had 0.1 ≤ ROAV <1 in the composite potato chips. This suggested that there was a significant difference in the key volatile flavor compounds between fresh-cut potato chips and composite potato chips. However, there was no significant difference in the key volatile flavor compounds in the composite potato chips prepared by the frying and baking processing methods.

**Table 6:** ROAV values of volatile flavor compounds in potato chips.

Count	Compound name	Order threshold (μg/kg)	ROAV				
			LS	SYS	SY	XSC	KBK
1	(E, E)-2,4-Decadienal	0.07	26.82	3.54	4.19	3.42	3.57
2	3-(methylsulfanyl)propanal-M	0.20	5.63	1.34	1.53	1.12	1.16
3	3-(methylsulfanyl)propanal-D	0.20	13.15	1.42	1.27	1.14	1.13
4	1-Nonanal	1.00	1.20	0.24	0.23	0.22	0.22
5	(E)-2-Butenal	0.02	32.88	26.56	26.72	11.17	12.89
6	Butanal	0.67	1.59	0.44	0.35	0.33	0.34
7	1-Hydroxy-2-propanone-M	0.08	22.95	3.04	3.18	2.82	3.27
8	1-Hydroxy-2-propanone-D	0.08	5.40	4.83	3.59	2.87	3.16
9	1-Octen-3-one	0.005	100.00	100.00	100.00	100.00	100.00
10	1-Penten-3-one-M	1.00	1.52	0.25	0.23	0.23	0.22
11	1-Penten-3-one-D	1.00	1.13	0.22	0.23	0.23	0.22
12	Acetic acid ethyl ester	0.10	6.79	2.43	3.63	2.39	2.29
13	1-Butanol-3-methyl-acetate-M	2.00	1.09	0.11	0.11	0.11	0.11

### 3.6 Principal component analysis (PCA) of different potato chips

**Figure 4:**
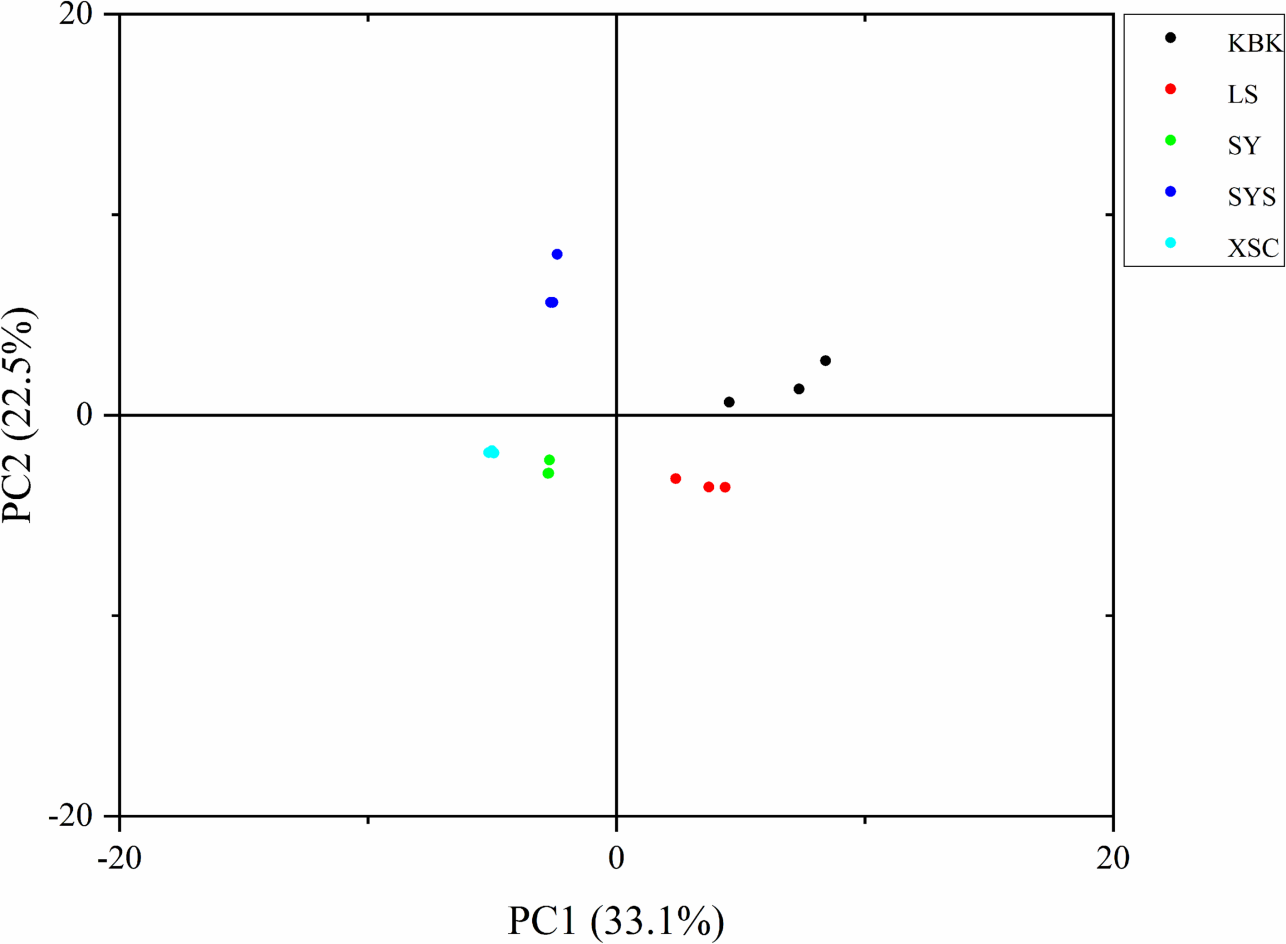
Principal component analysis (PCA) of different potato chips.

In Figure 4, the degree of differences between the potato chips can be visualized by PCA analysis of the volatile flavor compounds separated by GC-IMS (36). The first two principal components of potato chips, PC1 and PC2, were 33.1 and 22.5%, respectively, with a cumulative contribution of 55.6%. On PC1, LS, SY, and XSC were farther away from each other, which can better distinguish these samples. However, SYS and XSC, LS and KBK were closer, which were difficult to distinguish. But combined with PC2, the potato chips could be better distinguished. Overall, the long distance between the five potato chips and the lack of overlap in the images indicated that PCA analysis was able to effectively differentiate the odor changes of different types of potato chips.

### 3.7 Sensory evaluation of different potato chips

With respect to potato flavor, LS industrial fresh-cut fried potato chips received a higher rating. The potato flavors of the SYS homemade composite baked potato chips and SY industrial composite baked potato chips were similar. It suggested that fresh-cut potato chips had a stronger potato flavor. It was consistent with the findings of the potato chips’ volatile flavor compounds. The potato flavor of composite baked potato chips mainly came from potato powder or exogenously added fragrances. The deep-fried flavor of LS industrial fresh-cut fried potato chips and KBK industrial composite fried potato chips was more intense than other composite baked potato chips. They were favored by sensory evaluators, which was in agreement with the results of the determination of volatile flavor compounds of potato chips. However, due to their high fat content, the potato chips were also relatively heavy in fat flavor and greasiness. Additionally, the fried potato chips were susceptible to fats becoming rancid while being stored, which gave them a harrying flavor. In the sensory evaluation, SY industrial composite potato chips and SYS homemade composite baked potato chips were preferred overall. For producers, they are more willing to produce compound potato chips because they are convenient and controllable to produce potato chips. For consumers, using the baking method can reduce the fat content of potato chips, so consumers are more willing to accept the composite baked potato chips (see Table 7).

**Table 7:** Sensory evaluation of potato chips.

Items	LS	KBK	SYS	SY	XSC
Potato flavor	8.73 ± 0.47^a^	5.45 ± 1.29^c^	7.36 ± 1.29^ab^	7.55 ± 1.21^ab^	6.27 ± 2.00^bc^
Deep-fried flavor	6.73 ± 1.79^a^	6.73 ± 1.35^a^	6.36 ± 1.69^a^	5.55 ± 1.81^a^	5.45 ± 2.25^a^
Fatty flavor	6.73 ± 1.85^a^	6.55 ± 1.44^a^	6.09 ± 1.30^a^	5.91 ± 1.14^a^	5.82 ± 1.94^a^
Greasiness	7.09 ± 1.04^ab^	7.55 ± 1.21^a^	6.27 ± 1.01^b^	6.18 ± 1.08^b^	6.82 ± 1.54^ab^
Taste	6.82 ± 2.27^ab^	5.64 ± 2.11^b^	7.82 ± 0.98^a^	8.00 ± 1.10^a^	7.36 ± 1.80^a^
Overall preference	6.64 ± 1.12^ab^	6.45 ± 1.51^b^	7.55 ± 1.04^ab^	8.09 ± 0.83^a^	6.55 ± 2.16^b^

Means superscripted by different letters in the same row are significantly different at *p* < 0.05.

### 3.8 Correlation analysis

**Figure 5:**
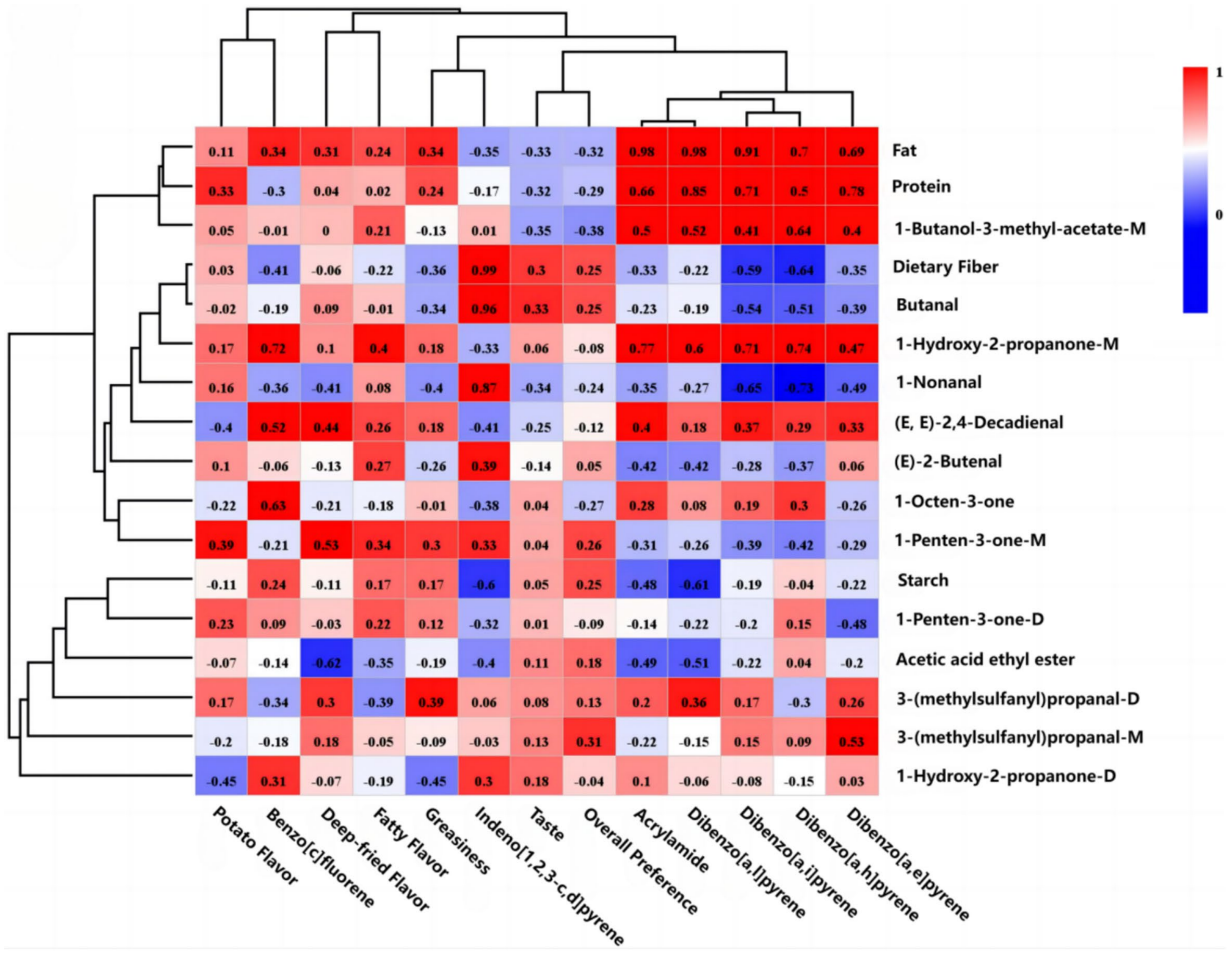
Pearson’s correlation heatmap showing physicochemical properties, sensory characteristics, and the key volatile flavor compounds of different potato chips. Note: Each color represents the correlation coefficient, with red and blue indicating positive and negative correlation, respectively.

A correlation heat map (Figure 5) between the physicochemical properties, sensory characteristics, and the key volatile flavor compounds showed a potential relationship (37). In sensory evaluation, potato flavor of potato chips was positively correlated with fat, protein, 1-hydroxy-2-propanone-M, 1-penten-3-one-M, 1-penten-3-one-D, and 3-(methylsulfanyl)propanal-D. The taste and overall preference of the potato chips were positively correlated with dietary fiber, starch, butanal, 1-penten-3-one-M, 3-(methylsulfanyl)propanal-M, 3-(methylsulfanyl)propanal-D, and acetic acid ethyl ester. This was mainly due to the fact that these flavor compounds contribute pleasant flavors. For example, acetic acid ethyl ester provided fruit aroma to potato chips. 3-(methylsulfanyl)propanal-D provided potato aroma to potato chips (15). Most of the aldehydes originate from the oxidation of fat by oxidation. The potato flavor is one of the reasons that contribute to consumer preference for fresh-cut potato chips. And the potato flavor in composite potato chips may be derived from whole potato flour or exogenously added flavors and fragrances. Fatty flavor, deep-fried flavor, and greasiness were positively correlated with fat, protein, (E, E)-2,4-decadienal, 1-hydroxy-2-propanone-M, and 1-penten-3-one-M due to the fact that these flavor compounds were mainly generated by oxidative degradation of fats and protein-related Maillard reactions (38). However, fat could make potato chips feel greasy, which reduced the sensory evaluation. So the taste and overall were negatively correlated with fat. At the same time, fat can cause harm to people’s bodies. In terms of health and sensory evaluation, consumers are more likely to accept composite baked potato chips with lower fat content.

Acrylamide was positively correlated with fat, protein, (E, E)-2,4-decadienal, 1-hydroxy-2-propanone-D, 1-hydroxy-2-propanone-M, 1-butanol-3-methyl-acetate-M, 1-octen-3-one, and 3-(methylsulfanyl)propanal-D. It was shown that acrylamide was mainly formed through the Maillard reaction. And most of the ketones were the products of the Maillard reaction, indicating that the generation of acrylamide could be reduced by controlling the Maillard reaction. PAHs in potato chips were mainly positively correlated with volatile compounds such as aldehydes and ketones originating from the oxidative degradation of fats. For example, dibenzo[a,l]pyrene, dibenzo[a,i]pyrene, dibenzo[a,h]pyrene, dibenzo[a,e]pyrene, and benzo[c]fluorene were positively correlated with (E, E)-2,4-decadienal and 1-hydroxy-2-propanone-M. The results showed that the PAHs in potato chips were positively correlated with the volatile flavor compounds that originated from fat oxidative degradation. Indeno[1,2,3-c,d]pyrene was positively correlated mainly with butanal, 1-nonanal, (E)-2-butenal, 1-penten-3-one-M, 3-(methylsulfanyl)propanal-D, and 1-hydroxy-2-propanone-D. Fat and protein in food are important factors that contribute to the formation of PAHs (21, 22), which can be reduced by changing the processing methods to reduce the fat and protein content, thus reducing the production of PAHs.

According to the preliminary market research, this study selected four commercial potato chip brands with high market acceptance within the shelf life, in contrast with the SYS homemade potato chips. Correlation between physicochemical properties, sensory characteristics, and key volatile flavor compounds is illustrated to provide a reference for subsequent formulation and preparation processes. However, the study has some limitations, focusing only on the five types of potato chips, which limits the generalizability of the results.

## 4 Conclusion

In this study, the differences in physicochemical properties, sensory characteristics, and volatile flavor compounds of five potato chips were investigated. A total of 57 volatile flavor substances were identified by GC-IMS. Seven key volatile flavor substances were common to the five potato chips, including (E, E)-2,4-decadienal, 3-(methylsulfanyl)propanal-M, 3-(methylsulfanyl)propanal-D, (E)-2-butenal, 1-hydroxy-2-propanone-M, 1-hydroxy-2-propanone-D, and 1-octen-3-one. In addition, the LS industrial fresh-cut fried potato chips were richer in key flavor compounds such as 1-nonanal, butanal, 1-penten-3-one-M, 1-penten-3-one-D, acetic acid ethyl ester, and 1-butanol-3-methyl-acetate-M. While the types of key volatile flavor compounds in LS industrial fresh-cut fried potato chips and other composite potato chips varied, the types of key volatile flavor compounds in composite fried and baked potato chips made with various formulations were consistent. In the sensory evaluation, SY industrial composite potato chips and SYS homemade composite baked potato chips were preferred overall. The fried potato chips had greater relative levels of harmful factors, but none of the potato chips included trans-fatty acids. Pearson correlation analysis revealed significant correlations between physicochemical properties, sensory characteristics, and key volatile flavor compounds. The correlation heatmap showed that the harmful factors in potato chips were mainly positively correlated with volatile compounds such as aldehydes and ketones originating from the oxidative degradation of fat. The study provided a reference for choosing appropriate process conditions in potato chip processing so that the safety of the food can be enhanced while obtaining consumer-preferred food flavors.

## Data Availability

The raw data supporting the conclusions of this article will be made available by the authors, without undue reservation.
